# Biochemical Neuroadaptations in the Rat Striatal Dopaminergic System after Prolonged Exposure to Methamphetamine Self-Administration

**DOI:** 10.3390/ijms231710092

**Published:** 2022-09-03

**Authors:** Subramaniam Jayanthi, Bruce Ladenheim, Patricia Sullivan, Michael T. McCoy, Irina N. Krasnova, David S. Goldstein, Jean Lud Cadet

**Affiliations:** 1 Molecular Neuropsychiatry Research Branch, NIDA Intramural Research Program, National Institutes of Health, Baltimore, MD 21224, USA; 2 Autonomic Medicine Section, NINDS Intramural Research Program, National Institutes of Health, Bethesda, MD 20892, USA; 3 Division for Research Capacity Building, NIGMS, National Institutes of Health, Bethesda, MD 20892, USA

**Keywords:** dopamine, dopamine metabolites, DOPAL, dopamine receptors, dorsal striatum, methamphetamine use disorder, parkinsonism

## Abstract

Perturbations in striatal dopamine (DA) homeostasis might underlie the behavioral and pathobiological consequences of METH use disorder in humans. To identify potential consequences of long-term METH exposure, we modeled the adverse consequence DSM criterion of substance use disorders by giving footshocks to rats that had escalated their intake of METH during a drug self-administration procedure. Next, DA D1 receptor antagonist, SCH23390 was injected. Thereafter, rats were euthanized to measure several indices of the striatal dopaminergic system. Footshocks split the METH rats into two phenotypes: (i) shock-sensitive that decreased their METH-intake and (ii) shock-resistant that continued their METH intake. SCH23390 caused substantial dose-dependent reduction of METH taking in both groups. Stopping SCH23390 caused re-emergence of compulsive METH taking in shock-resistant rats. Compulsive METH takers also exhibited greater incubation of METH seeking than non-compulsive rats during withdrawal from METH SA. Analyses of DA metabolism revealed non-significant decreases (about 35%) in DA levels in resistant and sensitive rats. However, striatal contents of the deaminated metabolites, DOPAL and DOPAC, were significantly increased in sensitive rats. VMAT2 and DAT protein levels were decreased in both phenotypes. Moreover, protein expression levels of the D1-like DA receptor, D5R, and D2-like DA receptors, D3R and D4R, were significantly decreased in the compulsive METH takers. Our results parallel findings in post-mortem striatal tissues of human METH users who develop Parkinsonism after long-term METH intake and support the use of this model to investigate potential therapeutic interventions for METH use disorder.

## 1. Introduction

Methamphetamine (METH) is a popular psychostimulant used worldwide [1,2,3,4,5]. In the United States, METH-associated overdose deaths increased nearly five-fold from 2012–2018 [6,7]. Comorbid METH-opioid use has also impacted the incidence of overdose deaths [8]. The clinical course of METH use disorder (MUD) includes increasing intake of the drug and repeated relapses during periods of abstinence even while patients are attending treatment programs [9,10,11]. Despite the popularity of METH use, there are, at present, no effective FDA-approved pharmacological interventions for MUD.

In order to develop beneficial therapeutic approaches against MUD, it is important to elucidate the biochemical bases of relapses to drug-taking behaviors using animal models of this neuropsychiatric disease [12,13,14]. Recently, our laboratory has employed footshocks that are contingently administered to rats that had escalated their METH intake. This approach has helped to separate rats into two groups based on their METH intake in the presence of punishment [15]. These groups have been labeled: (i) shock-resistant (SR, compulsive, vulnerable to METH addiction) and (ii) shock-sensitive (SS, abstinent, not vulnerable to addiction) groups [15,16,17,18,19]. This model was employed successfully to document important differences between the vulnerable and non-vulnerable phenotypes [15,16,17,18,19,20,21].

In the present study, we have expanded on the use of the model in order to identify potential perturbations in the striatal dopamine (DA) system because the accumulated evidence indicates that this structure is significantly impacted in human METH users [22,23,24,25]. Importantly, these perturbations appear to predict relapses in drug use [26]. In addition, dysregulation of DA homeostasis in the nigrostriatal system is thought to be mediators of the neurodegenerative processes that cause Parkinson’s disease (PD) [27,28] and might serve as substrates for the increased incidence of Parkinsonism reported in METH users who show a 165% higher risk index for the development of the disease than control groups [29,30]. Regrettably, despite the likely functional importance of striatal DA in METH use disorder and its neuropsychiatric consequences, there have been few studies that have investigated the long-term effects of extended METH self-administration (SA) on indices of dopaminergic system functions in animal models [31,32,33,34]. Importantly, no previous investigations have examined potential dysregulations of DA systems in shock-resistant and sensitive METH-taking rats.

The present study thus aimed to examine the effects of the selective DA D1-like receptor antagonist, SCH23390, on METH SA in rats that had been dichotomized to be compulsive or non-compulsive METH takers in the presence of footshock punishment. We chose to test the effects of this drug because the accumulated literature indicates that self-administration of psychostimulants including cocaine is dependent, in part, of stimulation of D1-like receptors because SCH23390 has been reported to suppress reinstatement of cocaine-seeking behavior [35,36]. Intra-striatal infusion of the drug can also reduce METH SA [37]. In addition to investigating the effects of the drug on METH SA, we used rat brain tissues to measure presynaptic and postsynaptic markers of the striatal dopaminergic system.

## 2. Results

### 2.1. The DA D1 Antagonist, SCH23390, Suppresses Compulsive METH Taking in the Presence of Contingent Footshock Punishment

**Figure 1:**
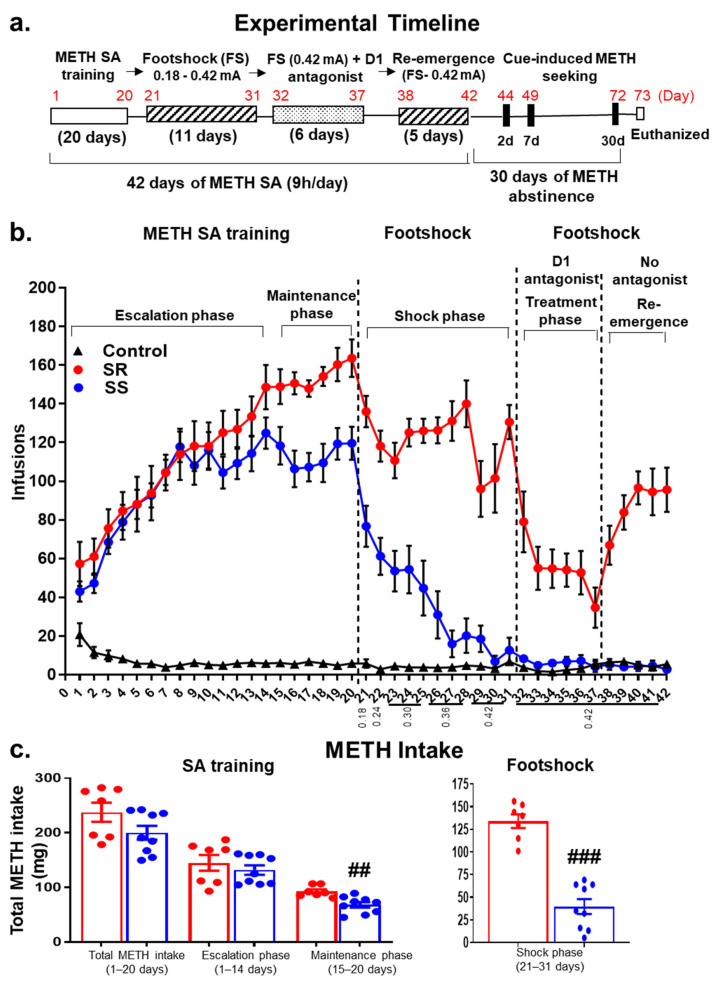
(**a**) Experimental timeline for METH SA, contingent footshocks, and administration of the D1 antagonist, SCH23390. (**b**) Patterns of METH (n = 16) or saline (CT, n = 8) SA in male Sprague–Dawley rats. Infusion patterns during 20 days of SA training include escalation (1–14 days), maintenance (15–20 days) and footshock (21–31 days) phases. During the shock phase, current intensity was increased gradually from 0.18 to 0.42 mA over 11 days. METH SA rats were segregated into two distinct phenotypes based on their response to contingent footshocks. The shock-resistant rats (SR, n = 7) showed less than 20% decreases in the average number of METH infusions from pre-shock levels despite the footshocks. The shock-sensitive rats (SS, n = 9) significantly reduced their METH intake. On the days of SCH23390 administration, rats were given the drug 30 min prior to the METH SA sessions. SCH23390 reduced the number of METH infusions (*p* < 0.0001) in both phenotypes. After stopping SCH23390 administration, SR, but not SS, rats returned to compulsive METH-taking behaviors. Data represent the number of daily infusions during 9 h of access to METH or saline (0.1 mg/kg/infusion). (**c**) Total METH intake by SR (n = 7) and SS (n = 9) rats during the 20 days of METH SA training followed by footshock (0.18–0.42 mA). The values represent means ± SEM. Key to statistics: ##, ### = *p* < 0.01, 0.001, comparison between SR and SS METH groups.

The experimental design, illustrated in Figure 1a, shows the timeline for METH SA and footshock experiments, as well as treatment of D1 antagonist (SCH23390) followed by the resurgence phase due to stopping SCH23390. Rats were trained to self-administer METH or saline for 20 days (Figure 1b). The groups were separated post facto based on their responses to footshocks as shock-resistant (SR) and -sensitive (SS) rats. The two-way ANOVA analysis for METH infusions included the between-subject factor of group (SR (n = 7), SS (n = 9), and CT (n = 6)) and the within-subject factor of SA session (training days 1–20). The analysis revealed significant effects of group (F (2, 21) = 93.67, *p* < 0.0001), training days (F (19, 399) = 43.62, *p* < 0.0001) and group x training days interaction (F (3, 90) = 2.758, *p* = 0.0469), thus indicating that all METH SA rats took METH during SA training.

The pattern of METH infusion during 20 days of SA training includes an “escalation phase” (1–14 days) and “maintenance phase” (15–20 days). As shown in Figure 1b, during the first 14 days of the escalation phase of METH. SA, SR and SS rats took similar amounts of METH, with no significant effects of the group (F (1, 14) = 0.68, *p* = 0.428). However, repeated measures ANOVA on maintenance phase (days 15–20) showed significant effects of the group (F (1, 14) = 13.40, *p* = 0.0026) and training days (F (5, 70) = 5.42, *p* = 0.0003). Post hoc analysis showed that the SS group differed significantly from the SR groups during the maintenance phase (*p* < 0.01) (Figure 1c, first panel). This is the first time that we have made the observation that rat Sprague–Dawley differed in their METH intake during the maintenance phase of drug SA since we had not observed similar differences in our previous studies [18,19,20]. However, these observations are consistent with the report that outbred male SD rats usually exhibit genetic and behavioral heterogeneity during responses to cues associated with food or drugs [38].

**Figure 2:**
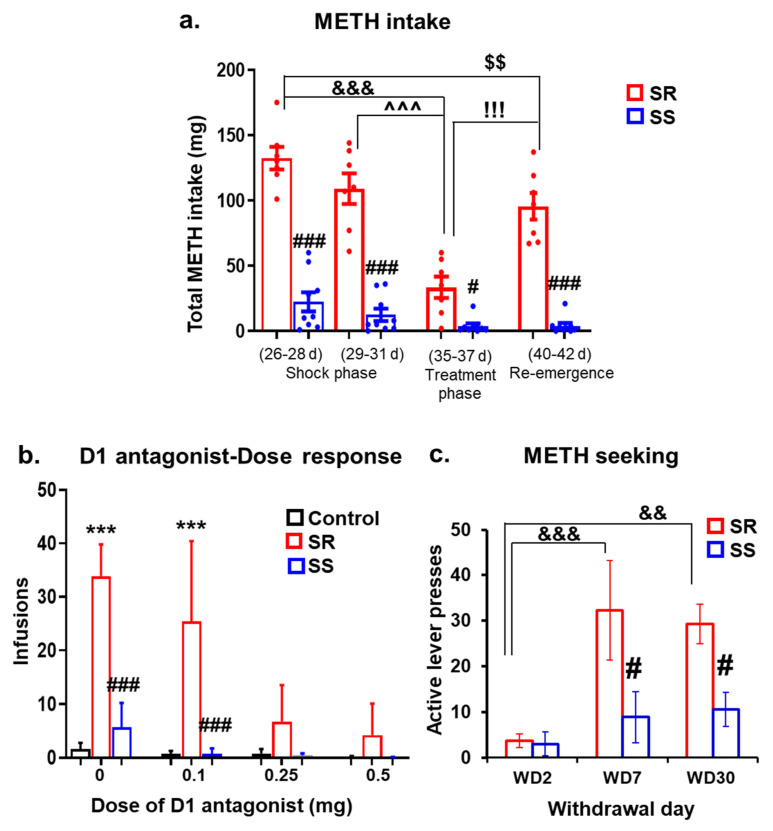
The DA antagonist, SCH23390, suppresses METH-taking behavior. (**a**) METH intake during footshocks and the effects of SCH23390 treatment and cessation of SCH23390 treatment. Increased intensity of footshocks reduced METH intake in SS but not in SR rats (# *p* < 0.05, ### *p* < 0.001, SR vs. SS). Administration of SCH23390 (35–37 d/days) before METH SA sessions significantly decreased METH intake in SR rats in comparison to days 26–28 (&&& *p* < 0.001) and days 29–31 (^^^ *p* < 0.001) of training plus contingent shocks. Stopping SCH23390 treatment led to re-emergence (days 40–42) of compulsive METH SA in comparison to SCH23390 treatment phase (!!! *p* < 0.001). Two-way repeated measures analysis for METH consumed included the between-subject factors (treatment groups, SR vs. SS) and within-subject factor (four intervals of training days, 26–28 d; 29–31 d; 35–37 d and 40–42 d), and their interactions. We found significant effects of treatment groups (F (1,14) = 134.40, *p* < 0.0001), training days (F (3,42) = 36.58, *p* < 0.0001) and the interaction of the two (F (3,42) = 19.04, *p* < 0.0001). Key to statistics: # *p* < 0.05; ### *p* < 0.001, comparison to respective SR group; &&& *p* < 0.001, comparison of SR rats between treatment phase (35–37 d) and 26–28 d of footshock; ^^^ *p* < 0.001, comparison of SR rats between treatment phase (35–37 d) and 29–31 d of footshock; $$ *p* < 0.01, comparison of SR rats between re-emergence phase (40–42 d) and 26–28 d of footshock; !!! *p* < 0.001, comparison of SR rats between re-emergence phase (40–42 d) and treatment phase (35–37 d). (**b**) Dose-related effects of SCH23390 on METH infusions. Intraperitoneal injections of increasing doses of SCH23390 caused dose-dependent decreases in the number of METH infusions (F (11,84) = 33.43, *p* < 0.0001), analysis of variance followed by Sidak’s post hoc test. The values represent means ± SEM. Key to statistics: *** *p* < 0.001 vs. control; ### *p* < 0.001 vs. SR. (**c**) Time-dependent cue-induced METH seeking in SR and SS rats. The figure shows lever presses on drug-associated (active) lever on withdrawal days 2 (WD2), 7 (WD7) and 30 (WD30) of METH withdrawal. SR and SS phenotypes show minimal cue-induced drug seeking behavior on WD2. However, SR, but not SS, rats showed significant cue-induced METH seeking at WD7 and WD30. Two-way ANOVA analysis included groups (SR, SS), withdrawal days (WD2, WD7 and WD30). There were significant effects of group (F (1, 14) = 6.36, *p* = 0.0244), withdrawal days (F (2, 28) = 9.52, *p* = 0.0007) and interactions (F (2, 28) = 3.583, *p* = 0.0412), indicating incubation of METH seeking over the withdrawal period. Post hoc tests (Sidak’s) showed greater responses at active levers by SR rats for both WD7 (*p* < 0.001) and WD30 (*p* < 0.01) compared to WD2, whereas SS rats showed no incubation. There were also greater responses in SR rats on WD7 (*p* < 0.05) and WD30 (*p* < 0.05) in comparison to SS rats. Data are presented as means ± SEM of number of active lever presses. Key to statistics: && *p* < 0.01, &&& *p* < 0.001, significantly different from WD2; # *p* < 0.05, significantly different from SR rats.

During the footshock punishment phase (11 days), shock intensity was increased gradually from 0.18 to 0.42 mA (Figure 1b). This response-contingent punishment caused significant decreases in METH infusions (F (10, 140) = 5.49, *p* < 0.0001), with SR rats continuing to lever press at a significantly high level and SS rats substantially reducing their METH intake. Criteria for assignments to SR and SS groups were as described in the Method Section. Bonferroni tests revealed significant differences in METH intake between SR and SS rats (*p* < 0.001) (Figure 1c, second panel) during the shock phase. Shock-induced segregation of the two groups could be observed on days 26–28 (0.36 mA), becoming quite prominent on days 29–31 (0.42 mA). At these intensities, SR rats took an average of 132.4 and 109.1 mg, whereas SS rats self-administered an average of 22.4 and 12.5 mg of METH, respectively (Figure 2a).

To test whether the DA D1 receptor antagonist could influence compulsive METH-taking behaviors, we administered various doses of SCH23390 for 6 days after the METH SA rats had separated into SR and SS groups. All the METH SA rats were given SCH23390 (0, 0.1, 0.25 and 0.5 mg/kg, i.p.), whereas the CT rats were treated with vehicle (saline, i.p.) 30 min prior to each SA session. Repeated measures ANOVA showed that SCH23390 caused significant dose-dependent decreases in the number of METH infusions in both SR and SS animals (Figure 2b, (F (4, 56) = 12.47, *p* < 0.0001)). The METH intake for SR rats plummeted from 109.1 mg prior to SCH23390 treatment to 33.5 mg after treatment while that of SS rats decreased from 12.5 to 3.8 mg before and after the inhibitor, respectively (Figure 2a).

Because we also wanted to know how long these suppressive effects of SCH23390 would last, we continued the METH SA procedure in the presence of footshocks after stopping the antagonist. Two-way ANOVA analysis for METH intake within subject factor of SA session, comparing METH intake during and after cessation of SCH23390 administration, showed significant differences in SR rats (F (9, 54) = 3.37, *p* = 0.0024) but not in SS rats (F (9, 72) = 1.72, *p* = 0.126). Specifically, METH intake increased in the SR, but not in the SS group, after stopping SCH23390 (see Figure 1b and Figure 2a). The fold differences in METH intake between SR and SS animals are shown in Figure S1.

### 2.2. Shock-Resistant Rats Exhibit Greater Incubation of METH Seeking Than Shock-Sensitive Rats after Prolonged METH Withdrawal

Figure 2c illustrates the differences in cue-induced lever pressing in SR and SS rats over several days of withdrawal from METH SA. Cue-induced active lever pressing was measured on withdrawal day 2 (WD2), WD7, and WD30 (Figure 2c). Two-way ANOVA revealed the main effects of withdrawal days (F (2, 28) = 9.52, *p* = 0.0007), group (SR vs. SS) (F (1, 14) = 6.36, *p* = 0.024), and significant interactions between withdrawal days x group (F (2, 28) = 3.58, *p* = 0.041). Post hoc tests showed greater responses of active lever pressing on WD7 and WD30 for SR rats compared to WD2 (Figure 2c). In addition, SR rats press the active lever at a higher clip than SS rats on WD7 (*p* < 0.01) and WD30 (*p* < 0.05). These observations are similar to our previous results [17,19,21]. There were, however, no significant differences in active lever pressing in SS rats on WD7 and WD30, consistent with the suggestion that consumption of larger quantities of METH is necessary for patients to relapse [39].

### 2.3. Withdrawal from METH SA Elicits Increased Dopamine Metabolism in the Dorsal Striatum of Shock-Sensitive Rats

**Figure 3:**
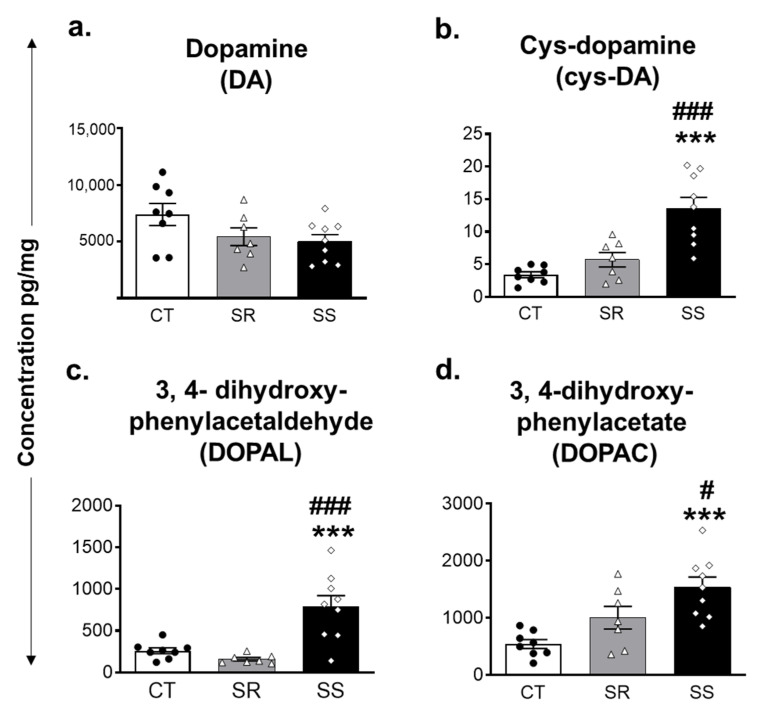
Levels of dopamine and its metabolites in the dorsal striatum. (**a**) DA, (**b**) cys-DA (**c**) DOPAL, and (**d**) DOPAC in SR, SS, and control rats. Key to statistics: *** *p* < 0.001, comparison control vs. SR and SS groups; # *p* < 0.05, ### *p* < 0.001, comparison between SR and SS groups. All values represent means ± SEM. CT (n = 8), control saline rats; SR (n = 7), shock-resistant, vulnerable, compulsive METH takers; SS (n = 9), shock-sensitive, non-vulnerable group.

As mentioned above, there is evidence for potential perturbations in the striatal DA system in human METH users [22,23,24,25]. These also appear to contribute to relapses to drug-taking behaviors [26]. Thus, we thought it important to characterize the effects of withdrawal from METH SA on the levels of DA and its metabolites in the dorsal striatum. DA levels showed a trend toward a decrease (~35%) in both SR and SS rats (Figure 3a) in comparison to CT rats. The product of spontaneous oxidation to DA quinone, cysteinyl DA (cys-DA), showed significant increases in sensitive (non-compulsive) rats in comparison to resistant (compulsive) and control rats (Figure 3b).

Post hoc analysis of the oxidative deamination DA products, namely, aldehyde 3,4-dihydroxyphenylacetaldehyde (DOPAL) and 3,4-dihydroxyphenylacetic acid (DOPAC) showed that sensitive rats had higher levels of DOPAL (F (2, 21) = 14.63, *p* = 0.0001) and DOPAC (F (2, 21) = 10.32, *p* = 0.0008) in comparison to resistant and control rats (Figure 3c,d).

### 2.4. Shock-Sensitive Rats Have Higher MAO-A Protein Levels Than Shock-Resistant and Control Rats

**Figure 4:**
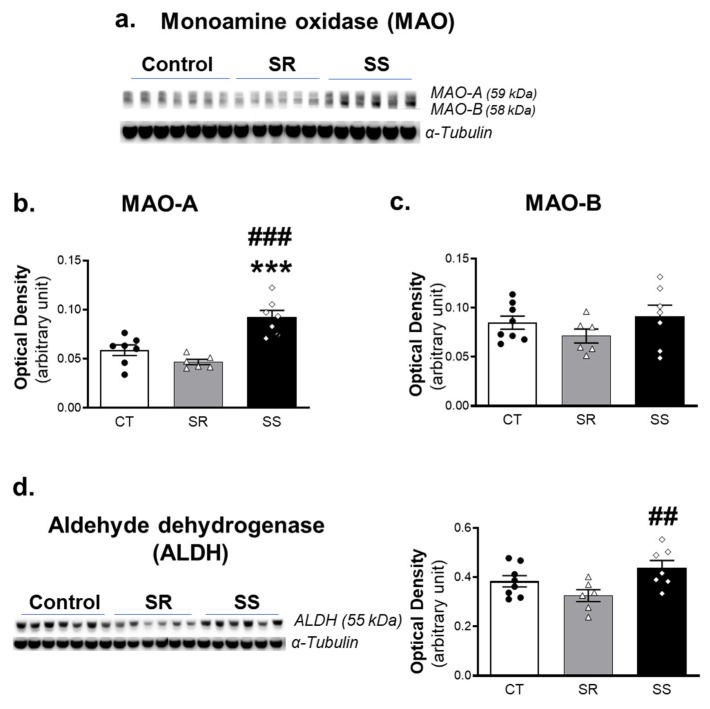
Protein levels of DA metabolizing enzymes. (**a**) Immunoblot analysis of MAO following withdrawal from METH self-administration. The anti-MAO antibody detected both MAO-A (59 kDa) and MAO-B (58 kDa). (**b**) Shock-sensitive rats exhibited higher levels of the MAO-A protein. (**c**) There were no differences in MAO-B levels between the three groups. (**d**) Western blot analysis of the ALDH enzyme in SR, SS, and control rats. ALDH protein levels were higher in the SS rats in comparison to SR rats. For quantification, the protein levels of MAO-A, MAO-B and ALDH were normalized to α-tubulin and then analyzed. Key to statistics: *** *p* < 0.001, comparison SR and SS vs. CT group; ## *p* < 0.01, ### *p* < 0.001, comparison between SR and SS groups. All values represent means ± SEM. CT (n = 8), control saline rats; SR (n = 7), shock-resistant, vulnerable, compulsive METH takers; SS (n = 9), shock-sensitive, non-vulnerable group.

Cytosolic DA in the rodent brain undergoes oxidative deamination by the FAD-dependent mitochondrial enzyme, monoamine oxidase-A (MAO-A) [40]. This reaction involves the conversion of DA to the aldehyde, DOPAL, with the generation of hydrogen peroxide. DOPAL is rapidly metabolized by aldehyde dehydrogenase (ALDH) to the acidic metabolite, DOPAC [41]. Because we observed increased levels of the deamination products in the non-compulsive/SS rats, we analyzed the protein expression of MAO-A and its isoenzyme, MAO-B, by Western blotting. We found that rats with increased deaminated products also exhibited higher levels of MAO-A protein levels in comparison to the other rats (F (2, 17) = 18.67, *p* < 0.0001) (Figure 4a,b). However, there were no significant differences (F (2, 18) = 1.219, *p* = 0.3189) in MAO-B protein levels (Figure 4a,c). Moreover, ALDH also showed significant higher levels in the sensitive rats in comparison to resistant rats (F (2, 18) = 4.455, *p* = 0.0268) (*p* < 0.01) (Figure 4d).

### 2.5. METH SA Caused Decreased VMAT2 and DAT Protein Levels in the Dorsal Striatum

**Figure 5:**
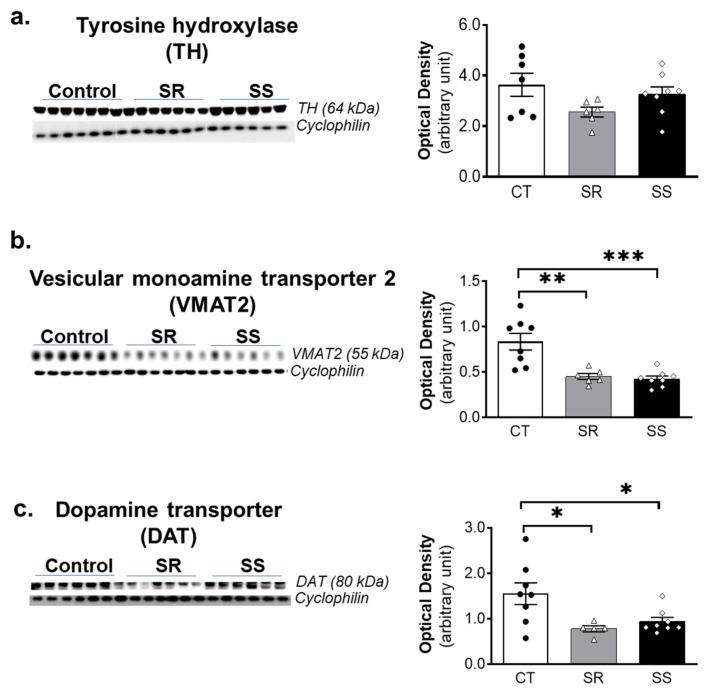
METH SA is associated with decreased VMAT2 and DAT protein levels in the dorsal striatum of both SR and SS rats. Protein levels of TH (**a**), VMAT2 (**b**) and DAT (**c**) were determined by Western blot analyses. The left panel shows the immunoblot images, and the right panel shows the quantitative measures. For quantification, the protein levels of TH, VMAT2 and DAT were normalized to cyclophilin and then analyzed. All values represent means ± SEM (n = 6–7 rats per group). Key to statistics: * *p* < 0.05, ** *p* < 0.01, *** *p* < 0.001, comparison to control saline group. The statistical analysis was performed using one-way ANOVA and Tukey’s post hoc tests.

METH administration has been reported to cause significant decreases in the levels of tyrosine hydroxylase (TH), vesicular monoamine transporter (VMAT), and dopamine transporter (DAT) [42]. Because these data were obtained from rodent studies that used large doses of METH injected by investigators, it was important to determine if these results could be translated to a model that better mimicked human use patterns. Toward that end, we measured striatal TH, VMAT2 and DAT protein levels by Western blot. These are reported in Figure 5. We found no significant changes in TH protein expression after METH SA (Figure 5a). In contrast, both SR and SS rats showed significant decreases in VMAT2 (F (2, 19) = 13.84, *p* = 0.0002) (Figure 5b) and in DAT (F (2, 18) = 5.605, *p* = 0.0128) (Figure 5c) protein levels in comparison to controls.

### 2.6. METH SA Caused Alterations in the Protein Expression of DA D1-like and D2-like Receptors in the Dorsal Striatum

**Figure 6:**
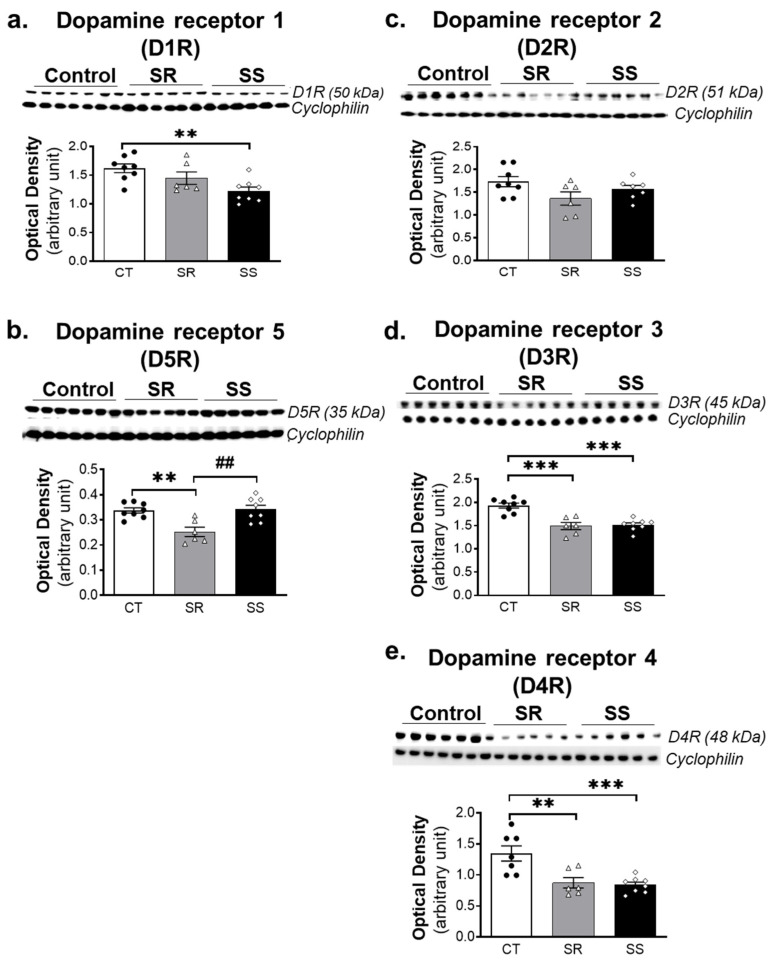
Protein levels of DA receptors are differentially impacted by METH SA in the dorsal striatum of rats. In the dorsal striatum, SS, but not SR, rats showed decreased D1R protein levels in comparison to the CT group (**a**). SR rats exhibited decreased D5R protein expression in comparison to CT and SS groups (**b**). There were no significant changes in D2R protein levels (**c**). Significant decreases in D3R and D4R protein levels were observed in both SR and SS rats (**d**,**e**). For quantification, the protein levels of D1R, D2R, D3R, D4R and D5R were normalized to cyclophilin and then analyzed. All values represent means ± SEM (n = 6–7 rats per group). Key to statistics: ** *p* < 0.01, *** *p* < 0.001, comparison to control saline group; ## *p* < 0.01, comparison to SR groups. The statistical analysis was performed using one-way ANOVA and Tukey’s post hoc tests.

In addition to measuring the impact of METH SA on markers of DA synthesis, metabolism, storage, and uptake mechanisms, we sought to determine if the drug might have impacted the expression of DA receptors that are highly expressed in intrinsic striatal neurons [43,44,45]. The effects of METH SA on striatal expression of D1-like (D1 and D5) and D2-like (D2, D3 and D4) receptor subtypes are shown in Figure 6. D1R showed significant decreases (F (2, 19) = 6.0, *p* = 0.0075) in the SS rats in comparison with the CT group (Figure 6a), whereas D5R showed significant decreases (F (2, 19) = 9.946, *p* = 0.0011) in the SR rats in comparison to CT and SS rats (Figure 6b). There were no significant changes in protein levels of D2R in SR and SS rats (Figure 6c). However, D3R (F (2, 19) = 19.81, *p* < 0.0001) and D4R (F (2, 19) = 10.73, *p* = 0.0009) showed significant decreases in protein levels in both SR and SS rats in comparison with the CT group.

## 3. Discussion

The present study documents the suppressing effects of the DA D1-like receptor antagonist on METH SA in rats that had been split into compulsive and non-compulsive drug takers after the application of footshocks. These results are consistent with those of Avchalumov et al. (2020) [37] who had reported that intrastriatal injections of SCH23390 could reduce METH SA. This is the first demonstration that stopping SCH23390 led to re-emergence of compulsive METH taking, even in the presence of contingent footshocks. Consistent with our previous publications [15,17,20], shock-resistant rats showed greater incubation of METH seeking than non-compulsive rats at withdrawal day 30, suggesting potential differences in biochemical neuroadaptations related to the dopaminergic system of these two phenotypes.

Biochemical analyses of DA metabolism in the dorsal striatum revealed no significant changes in DA content between the SR and SS groups, with small decreases (35%) in both METH SA phenotypes. These results suggest that all the animals had taken enough METH during the training phase to cause a partial depletion of striatal DA. The lower levels of total DA might be related to METH-induced abnormalities in vesicular DA storage with subsequent DA release into the terminal cytoplasm followed by spontaneous and enzymatic DA degradation. Furthermore, the present results are, in part, consistent with those of Krasnova et al. (2010) [32], who had reported significant decreases in striatal DA levels after prolonged exposure to METH. Those authors [32] had used METH SA for 15 h/day in their study, whereas we used 9 h/day in the present study. They also measured DA levels after 14 days of withdrawal, whereas we measured DA after 30 days of withdrawal. These differences in timing for DA analyses suggest the possibility that the levels of striatal DA might have recovered during one month of withdrawal used in the present study.

In contrast to the non-significant decreases in striatal DA levels, we found significant differences in the levels of oxidative products of DA metabolism in the striatum. Specifically, Cys-DA, DOPAL and DOPAC levels were higher in the sensitive rats in comparison to the SR and control rats, thus suggesting increased DA metabolism in that group, as suggested in the previous paragraph. The observed changes in these DA metabolites, together with increased Cys-DA levels, are consistent with a shift from vesicular uptake to increased cytoplasmic content followed by spontaneous and enzymatic oxidation.

These observations had suggested that there might also be potential differences in the expression of the enzymes that catalyze the metabolism of DA to DOPAL and then to DOPAC in the METH SA rats. The observations of increased MAO-A and ALDH protein levels in the sensitive in comparison to the resistant rats support this notion. The presumed increase in this two-step enzymatic sequence of MAO-A and ALDH might constitute an adaptive process in an attempt to reduce cytoplasmic DA levels in the SS group. It will be of interest to determine whether humans who are less likely to meet criteria for METH use disorder express specific MAO gene polymorphisms that enhance DA metabolism during intake of the drug. This idea is supported by recent findings of an association between cocaine and opioid use disorders with a MAO-B variable number tandem repeat (VNTR) polymorphism [46]. There is also an association between heroin dependence and MAO-A VNTR polymorphisms in male heroin users [47].

Previous preclinical and clinical studies had documented significant decreases in the expression of striatal TH, VMAT2, and DAT proteins after METH administration [42]. In the present study, we found that both resistant and sensitive rats had significant decreases in striatal VMAT2 and DAT protein levels. Those observations are consistent with data obtained after several days of withdrawal from METH SA in rats [32,48,49,50]. Those data are also consonant with observations in METH-using subjects that had been abstinent for at least 3 months [23,51,52]. Post-mortem studies using quantitative Western blotting have also identified decreased striatal DAT and VMAT2 protein levels in the striatum of chronic METH users [25]. A similar reduction in DAT immunohistochemistry was observed in putamen of chronic METH users along with caspase-3 activation, an indicator of neuronal apoptotic cell death [53]. Although we did not present histological evidence for cellular demise in the present study, when taken together with the published literature, our results indicate that the amount of METH consumed during the METH SA phase was sufficient enough to cause damage to intracellular vesicles secondary to METH-induced displacement of DA from vesicular pools and increased production of DA-dependent reactive oxygen species (ROS) that caused damage to striatal DA terminals [42,54]. This reasoning is consistent with previous preclinical observations documenting the fact that mice treated with a neurotoxic regimen of METH (4 × 10 mg/kg) showed significant loss of the terminal markers, TH and DAT, in the dorsal striatum in studies using immunoblotting and immunohistochemical measures [55]. The loss of terminal markers was attenuated in VMAT2-overexpressing mice [55,56]. Importantly, an opposite effect was observed in mutant mice that expressed only 5–10% of VMAT2 in comparison to wild-type animals [57]. Mice with low VMAT2 expression also showed massive argyrophilic deposits in the striatum after METH, thus indicating that VMAT2 is an important mediator of METH-induced neurodegeneration [57]. Taken together, all these studies that have previously provided both immunoblotting and immunohistochemical evidence of METH-induced DA terminal loss are consistent with our present observations after chronic METH self-administration.

DA exerts its action via interactions with D1- and D2-like DA receptors [58,59,60] that are highly expressed in the brain [61,62]. The decreased expression of D1R in the dorsal striatum of sensitive rats might be related to the increased DA metabolism observed in these rats (see Figure 3c,d and Figure 6a). In contrast, there were significant decreases in striatal D5R protein levels in the compulsive METH-taking rats. DA D1-like receptor downregulation observed in these groups might be compensatory consequences of METH-induced increases in DA in the synaptic cleft and increased DA interactions with these receptors. Importantly, the observations that the protein expression of these two receptors is differentially impacted in the two groups add support to the conclusion of previous reports that D1R and D5R are dissimilarly regulated during different brain functions and pathological states [63,64]. For example, D5R knock-out (KO) mice are reported to be more active than D1R KO mice after chronic cocaine injections [65]. In addition, D5R KO mice showed greater sensitivity to acute METH challenges by displaying greater ambulatory activity [66]. Taken together, these observations suggest the need for the development of pharmacological agents that are more receptor specific in order to help dissect the different contributions of these two D1-like receptors in the brain. Analysis of the effects of METH on DA D2-like receptors did not reveal any significant changes in striatal D2R expression in the SR and SS rats. However, there were significant decreases in D3R and D4R protein levels in both METH SA phenotypes. Together, these results suggest that striatal D3- and D4-containing neurons might be more involved in the long-term effects of METH than D2-containing neurons. These will need to be further investigated.

It is of interest to discuss the observations of decreased DAT and VMAT2 protein levels in both SR and SS rats in relations to previous data reported in the brains of human METH users [67]. Taken together, the significant decreases in DAT and VMAT2 as well as the small decreases in DA levels suggest that METH-induced oxidative processes that began in the striatum might have led to progressive changes not only in terminal areas but also retrograde damage that might eventually lead to disruption of cell bodies located in the nigrostriatal system [28,68,69]. This suggestion might provide partial explanation for the report of increased prevalence of Parkinsonism in human METH users [29,30,70]. More studies are needed to further characterize the status of DA systems in human METH users who present with signs and symptoms of Parkinsonism. Finally, future pre-clinical studies using other DA–ergic brain regions are necessary to assess similar or different responses to METH SA.

## 4. Materials and Methods

### 4.1. Animals

Male Sprague–Dawley rats (Charles River Labs, Raleigh, NC, USA) weighing 350–400 g were used. Rats were group-housed and habituated for 7–15 days prior to surgery. All rats were maintained on a 12 h reverse-light dark cycle with food and water available ad libitum. Prior to the start of the surgery, each rat was handled 2–3 min daily for at least 5 days. All animal procedures in the study were performed in accordance with the Guide for the Care and Use of Laboratory Animals- Eighth edition (ISBN 0-309-05377-3) and were approved by the National Institute on Drug Abuse Animal Care and Use Committee.

### 4.2. Drugs

(+)-Methamphetamine HCl (METH) (National Institute of Drug Abuse Pharmacy, Baltimore, MD, USA) was dissolved in 0.9% NaCl at a concentration of 0.1 mg/mL. R (+)-SCH-23390 hydrochloride (Research Biochemicals, Natick, MA, USA), was mixed in saline (0.9% sodium chloride) and injected intraperitoneally (i.p.) at a volume of 1 mL/kg. All drug doses were based on the salt form of the drug.

### 4.3. Apparatus

The experiments were conducted in operant chamber boxes (25 × 27 × 30 cm). Each box has two levers located 9 cm above the floor, but only one lever (an “active”, retractable lever) activates the infusion pump (Med Associates Inc., St. Albans, VT, USA). METH-HCL was dissolved in sterile saline at a concentration 0.1 mg/kg/infusion and loaded into syringes. Each syringe was mounted above infusion pumps and connected via a rotating “swivel” to a back mounted cannula, which finally led to the jugular catheter of the rat. Swivel apparatuses are sufficiently long enough to allow rats to move freely in the behavioral chamber. Data collection and programming were conducted using PC computers with a Med-PC interface (Med Associates, Inc., St. Albans, VT, USA).

### 4.4. Intravenous Catheter Implantation Surgery

Animals were deeply anesthetized with ketamine and xylazine (50 and 5 mg/kg, i.p., respectively) (NIDA pharmacy, Baltimore, MD, USA) and an indwelling catheter (SIA, Infusion Technologies, Lake Villa, IL, USA) was surgically implanted into the right jugular vein. The proximal end of the catheter was secured to the vein with surgical silk sutures and passed subcutaneously to the top of the back, where it exited into a connector (modified 22-gauge cannula, P1 Technologies, Roanoke, VA, USA). Buprenorphine (0.1 mg/kg, s.c.) was injected to relieve pain after surgery. Animals were allowed to recover for 5–7 days before METH SA training. During the recovery, training, and punishment phases of the experiment, catheters were flushed every other day with the antibiotic gentamicin (5 mg/mL) (Covetrus, Dublin, OH, USA) to prevent catheter blockages and infections. Usually, if a blockage is observed during flushing of a catheter, a second one would have been implanted in the left jugular vein, and the experiment would have been resumed following a 3 d recovery period from surgery. However, no re-implantation was necessary during this experiment.

### 4.5. METH Self-Administration and Effects of DA D1 Receptor Antagonist (SCH23390)

The behavioral procedure consisted of 4 phases: (i) METH self-administration training (days 1–20), (ii) contingent foot-shock punishment during METH self-administration (days 21–31), (iii) DA D1 receptor antagonist treatment during METH self-administration (days 32–37), and (iv) resurgence phase after stopping the administration of the DA D1 antagonist (days 38–42).

#### 4.5.1. Self-Administration (SA) Training Phase (Days 1–20)

Rats were randomly divided into two groups: (1) saline (n = 8) and (2) METH (0.1 mg/kg/infusion) (n = 16). On each training day, rats were trained to lever press on a fixed-ratio-1 (FR1) schedule for three 3 h daily sessions (total 9 h/d) with 30 min off intervals in between each session. This procedure lasted for 20 days.

The insertion of the active lever and the illumination of a red house light marked the beginning of each SA session. At the end of each 3 h session, the house light was turned off, and the active lever was retracted. For all rats, we recorded the number of infusions, active lever presses, and inactive lever presses.

During training, the rats lived in the SA chambers with free access to food and water. During the session, each press on the active lever resulted in an infusion delivered as a volume of 0.065 mL during a 2–3 s period, accompanied by a 5 s compound tone-light stimulus. After each infusion, there was a 20 s timeout infusion free period. This interval was designed to prevent drug overdose due to non-stop administration of METH. Responses on the inactive lever were also recorded but were not followed by METH infusions. Rat weights were monitored daily.

#### 4.5.2. Footshock Phase (Days 21–31)

After 20 days of METH SA, rats achieved stable responding, during which time the set criterion reached less than 10% variation over the last three SA training days. Thereafter, all METH SA rats received a contingent delivery of a 0.5 s footshock through the grid floor for 50% of the reinforced lever-presses. We set the initial footshock at 0.18 mA and increased the shock intensity by 0.06 mA up to a final current of 0.42 mA. This progression of footshock intensity occurred over a total of 11 punishment days. This model has been successfully used in previous publications from this laboratory [15,16,17,18,19,20,71].

Contingent footshocks segregated the animals into two phenotypes. One phenotype consisted of shock-resistant (SR, n = 7) rats that continued to compulsively press the lever for METH, whereas the other groups consisted of shock-sensitive (SS, n = 9) animals that had progressively decreased their METH intake in responses to the shocks. We included rats in the SR group if they showed less than a 40% decrease in the number of METH infusions by comparing the intake of the last 3 days of pre-shock to that of the last 3 days of the footshock phase (0.42 mA). SS rats had to show more than 60% suppression of drug infusions relative to pre-shock levels.

#### 4.5.3. DA D1 Antagonist (SCH23390) Treatment Phase (Days 32–37)

Next, we used intraperitoneal (i.p.) injections of different doses of a DA D1 receptor (D1R) antagonist, SCH23390 to measure its effects on METH SA. Both saline and METH self-administering rats received injections of SCH 23390 (0, 0.1, 0.25 and 0.5 mg/kg) following a Latin-square design at training days 32–37. All SCH23390 injections were given 30 min before each behavioral session. Contingent footshocks were also applied during that period with an intensity of 0.42 mA.

#### 4.5.4. Resurgence Phase (Training Days 38–42)

After investigating the effects of SCH23390, we stopped the drug and continued the behavioral experiment in the presence of contingent footshocks at the 0.42 mA intensity. We ran that phase of the experiment for 5 days.

### 4.6. Withdrawal

After the training, punishment, D1 antagonist treatment and resurgence phase, rats were relocated to the animal vivarium and individually housed with no access to METH. Rats had access to food and water ad libitum, and intravenous catheters were covered using dust caps (P1 Technologies, Roanoke, VA, USA). On days 2, 7 and 30 of withdrawal (WD), cue-induced drug seeking was assessed in all animals. For these tests, rats were brought back into their respective SA chambers in the morning. Each test consisted of a single 3 h session during which presses on the “active’’ lever resulted in contingent demonstration of the tone and light cues previous paired with METH. However, no METH was available during these tests. Cue-induced drug seeking behavior was examined using a within-subject design such that all rats tested on day 2 were also tested on day 7 and day 30 of withdrawal. Animals were euthanized one day after the third cue-induced METH seeking test.

### 4.7. Measurement of Dopamine and Metabolites

The levels of dopamine and its metabolites were measured using liquid chromatography with electrochemical detection (LCED) after batch alumina extraction, as described previously [41,72,73,74].

Striatal tissues were dissected out from both hemispheres and quickly frozen. Tissues from one hemisphere were homogenized using a Branson sonifier 150 in a solution of 20:80 of 0.035 M phosphoric acid: 0.2 M acetic acid using a ratio of 50 mg tissue to 250 μL of 20:80. The supernatant was assayed by batch alumina extraction followed by LCED with Waters 515 pump, Water 717 autosampler (Waters Corporation, Milford, MA, USA) and ESA Choulochem 3 electrochemical detector (ESA, Inc., Chelmsford, MA, USA) with series electrochemical detection, Cera column temperature controller 250 (Cera, Inc., Baldwin Park, CA, USA) set to 18 degrees using a Spheri-5 RP-18, 5 μm, 30 × 4.6 mm guard column (Perkin Elmer, Waltham, MA, USA, No. 07110013) and Bio-advantage C18, 5 µm, 120 Å, 4.6 × 250 mm analytic column (Thomson Instruments, Chantily, VA, USA, No. BA400-046250). The mobile phase consisted of 13.8 g monobasic sodium phosphate, 64 mg octane sulfonic acid, 50 mg EDTA, and 25–30 mL acetonitrile in 1 L of HPLC-grade water, adjust pH to 3.15–3.25 using 85% phosphoric acid. Concentrations of dopamine and its metabolites in the dorsal striatum were expressed in units of picomoles per milligram per weight.

### 4.8. Western Blot

Dorsal striatal tissues from the other hemisphere were homogenized using 10 mM Tris HCl, 150 mM NaCl, pH 7.5 in the presence of 1% Nonidet P-40 (NP-40) protein and phosphatase inhibitor cocktails (Sigma, St. Louis, MO, USA). Total protein concentrations were quantified using BCA assay (Thermofisher Scientific, Waltham, MA, USA). Then, 20 μg of soluble protein lysate was prepared in solutions that contained Laemmli buffer and 5% β-Mercaptoethanol. Samples were then boiled and resolved using NuPage 10% Bis-Tris Protein Gels (ThermoFisher Scientific, Waltham, MA, USA). Proteins were electrophoretically transferred onto PVDF membranes (Bio-Rad, Hercules, CA, USA). Membranes were blocked with 5% BSA in TBST and incubated overnight with primary antibodies at dilutions described by the manufacturer. Primary rabbit polyclonal antibodies including anti-D1R (1:1000; # PAS-27172); anti-D3R (1:1000; # PAS-79170); anti-D4R (1:1000; # PAS-104385); anti-D5R (1:1000; # 720284) were purchased from ThermoFisher Scientific (Waltham, MA, USA). Anti-ALDH1 (1:1000; # 15910-1-AP) and anti-D2R (1:1000; # 55084-1-AP) was purchased from ProteinTech Group, Inc. (Rosemont, IL, USA). Anti-DAT (1:1000; # AB1591P), anti-VMAT2 (1:1000; # AB1598P) anti-TH (1:1000; # 657012) and anti-α-tubulin (1:10,000; # T6074) was purchased from MilliporeSigma (Burlington, MA, USA). Anti- MAO A+B (1:1000; # BS-11890R) was purchased from Bioss Antibodies Inc. (Woburn, MA, USA). Cyclophillin rabbit polyclonal antibody (1:10,000, AB16045). was purchased from Abcam (Waltham, MA, USA). The antibodies revealed bands at the expected molecular weights for all proteins. Anti-rabbit HRP (1:6000 #7074) and anti-mouse HRP (1:6000 #7076) secondary antibodies were purchased from Cell Signaling Technologies (Danvers, MA, USA). After secondary antibody incubation, ECL Clarity (Bio-Rad, Hercules, CA, USA) was used to detect bands on ChemiDoc Touch Imaging System (Bio-Rad. Hercules, CA, USA), and intensities were measured with Image Lab 6.0 version (Bio-Rad, Hercules, CA, USA) software.

### 4.9. Statistical Analysis

Behavioral data were analyzed with the statistical program, GraphPad Prism (version 9, GraphPad software, La Jolla, CA, USA). To understand the nature of interactions further, 2-way ANOVA with repeated-measures analyses were also undertaken to compare the groups. Variables were numbers of METH infusions on training days, between-subject factors (CT, SR, and SS), and within-subject factor SA days (training days 1–20), and their interactions. Bonferroni post hoc tests were used to reveal the significant differences. METH seeking data were also analyzed using 2-way ANOVA with repeated-measures followed by Sidak’s post hoc test, with variables being group (CT vs. SR or SS) and withdrawal days (WD2, WD7 and WD30). The micro-dialysis and Western blot data were analyzed by one-way ANOVA followed by Tukey’s post hoc test using GraphPad Prism (Version 9.4.1, GraphPad software (San Diego, CA, USA)). The null hypothesis was rejected at *p* < 0.05.

## 5. Conclusions

**Figure 7:**
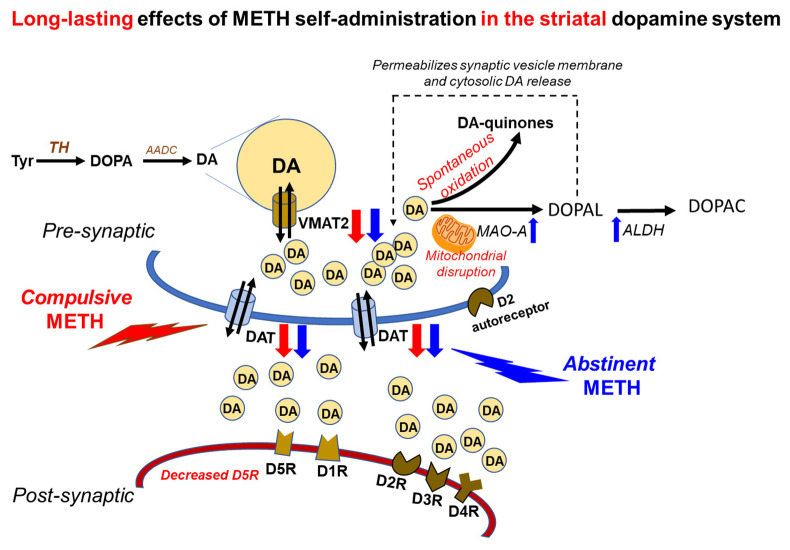
Schema illustrating potential mechanisms for the observed changes in the striatal dopaminergic system of METH self-administering rats. METH SA is accompanied by increased release of DA in dopaminergic terminals followed by DA release into the synaptic cleft where DA interacts with DA receptors. Increased cytosolic DA release is associated with enzymatic and non-enzymatic formation of reactive oxygen species known to cause significant decreases in VMAT2 and DAT protein levels as observed in both SR and SS rats herein. The differential expression of post-synaptic DA D1-like receptors (D1R and D5R) supports the notion that these genes might be differentially regulated. The decreased expression of the D2-like receptors, D3R and D4R, indicates that they might have undergone similar molecular adaptations in the presence of high levels of DA released in the dorsal striatum during METH SA.

In summary, the present study has documented a significant involvement of striatal DA system in the effects of compulsive METH taking in a manner consistent with the clinical literature that implicates disturbed DA dynamics in patients with METH use disorder [22,23,24,25,26]. Figure 7 provides a schema that represents potential DA-related mechanisms that might underlie compulsive METH-seeking behavior. The observations that the DA D1-like receptor antagonist blocks METH SA suppressed METH-taking behaviors suggest the involvement of D1-like receptors in the maintenance of METH taking, with the D5-like appearing to play a more important role, given the downregulation of the D5 receptor observed in the shock-resistant rats. This suggestion will need to be validated in METH SA experiments in rats that had undergone specific genetic manipulations of the expression of each subtype of DA receptors in their brain. The evidence of increased DA metabolism observed in the shock-sensitive rats implicates increased DA enzymatic breakdown in animals that decreased their METH intake in response to footshocks. The potential clinical relevance of the latter observations remains to be investigated in order to identify potential MAO gene polymorphisms that might be relevant to the clinical presentation and course of METH use disorder in humans. Experiments that measure the extravesicular release of DA in the striatum are also needed.

## Data Availability

Not applicable.

## Supplementary Materials

The following supporting information can be downloaded at: https://www.mdpi.com/article/10.3390/ijms231710092/s1.

## Abbreviations

ALDH, aldehyde dehydrogenase; Cys-DA, cysteinyl dopamine; DA, dopamine; DAT, dopamine transporter; DOPAC, 3,4-dihydroxyphenylacetic acid; DOPAL, 3,4-dihydroxyphenyl acetaldehyde; hr, hour; KO, knock-out; LCED, liquid chromatography with electrochemical detection; METH, methamphetamine; MUD, METH use disorder; MAO, monoamine oxidase; PD, Parkinson’s disease; SA, self-administration; SR, shock-resistant; SS, shock-sensitive; SD, Sprague–Dawley; TH, tyrosine hydroxylase; VNTR, variable number tandem repeat; VMAT2, vesicular monoamine transporter; WD, withdrawal day
