# Supplementary material for: Biochemical Neuroadaptations in the Rat Striatal Dopaminergic System after Prolonged Exposure to Methamphetamine Self-Administration
Source: Int J Mol Sci. 2022 Sep 3;23(17):10092. doi: 10.3390/ijms231710092 (PMC9456063; doi:10.3390/ijms231710092)
Supplement: Supplementary file 1 [file ijms-23-10092-s001.zip › ijms-1876988-supplementary.pptx]

## Slide 1
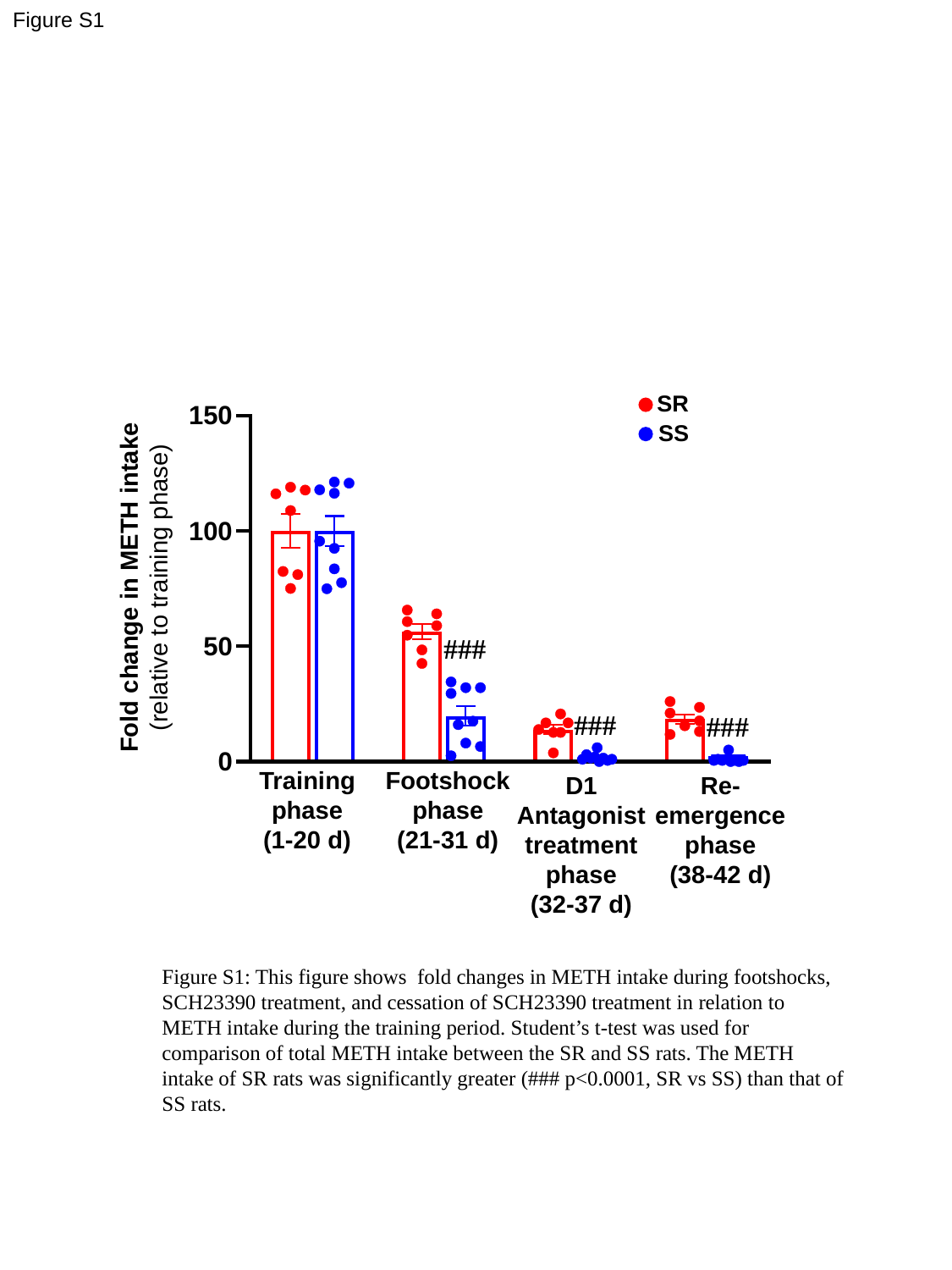

Figure S1
SR
SS
Fold change in METH intake
(relative to training phase)
###
###
###
Training phase
(1-20 d)
Footshock phase
(21-31 d)
D1 Antagonist treatment phase
(32-37 d)
Re-emergencephase
(38-42 d)
Figure S1: This figure shows fold changes in METH intake during footshocks, SCH23390 treatment, and cessation of SCH23390 treatment in relation to METH intake during the training period. Student’s t-test was used for comparison of total METH intake between the SR and SS rats. The METH intake of SR rats was significantly greater (### p<0.0001, SR vs SS) than that of SS rats.
